# Trace Oxygen Affects Osmium Redox Polymer Synthesis for Wired Enzymatic Biosensors

**DOI:** 10.1149/1945-7111/ac42a0

**Published:** 2022-01-18

**Authors:** Margaret C. Calhoun, Christopher D. Stachurski, Sara L. Winn, Evan A. Gizzie, Aaron W. Daniel, Nathan D. Schley, David E. Cliffel

**Affiliations:** Department of Chemistry, Vanderbilt University, Nashville, Tennessee 37235-1822, United States of America

## Abstract

Electrochemical sensors that utilize enzymes are a sensitive, inexpensive means of detecting biologically relevant analytes. These sensors are categorized based on their construction and method of signal transport. Type I sensors consist of a crosslinked enzyme on an electrode surface and are potentially subject to interference from byproducts and other biological analytes. However, type II sensors help alleviate this problem with the addition of a redox polymer layer that assists in signal transduction, thus minimizing interferences. An osmium-loaded poly(vinylimidazole) polymer (Os-PVI) is commonly used with successful results, and when combined with an enzyme yields a type II sensor. Our initial attempts at the synthesis of this polymer resulted in an unexpected osmium precursor, which had fluorescent and redox properties that did not match with the desired Os-PVI polymer. Careful exclusion of oxygen during the Os complex precursor synthesis was necessary to avoid this unexpected oxygen containing Os-precursor, which had been seen previously in mass spectrometry studies. All precursors and osmium polymers were characterized with ^1^H NMR, fluorescence, mass spectrometry, and cyclic voltammetry to provide a better understanding of these compounds and assist in the building of new sensors.

Biosensors are commonly utilized throughout medical, environmental, and food safety applications.^1,2^ Enzyme-based electrodes are a leading platform for these uses. The electrochemical enzyme biosensor is comprised of an enzyme immobilized onto an electrode surface, and when the substrate turns over at the enzyme, a redox co-factor is required to assist the enzyme in transforming the substrate. Since these redox co-factors must be recycled for another enzymatic reaction to occur, a mediator is typically used to recharge these co-factors.

Based on the electrochemical mediator used to recharge the enzymatic co-factor, the enzyme biosensor can be broadly classified as either Type I (oxygen mediated) or Type II (redox polymer mediated) (Fig. 1). Both types of sensors are in use today commercially, clinically, and in research. In a Type I biosensor, the electrons released by the enzyme-substrate turnover are accepted by a small molecule redox couple that diffuses to the underlying electrode. A common Type I example is oxidase enzyme electrodes, where oxygen that is naturally dissolved in water recharges the co-factors by accepting the electrons and becoming hydrogen peroxide. Hydrogen peroxide is collected by the underlying electrode set to a potential to oxidize the peroxide, producing a current. Different amounts of substrate can be exposed to the enzyme, producing different amounts of corresponding current that are recorded to create a calibration curve. Once this has been established, an unknown concentration of substrate can be detected.

To eliminate the need for the reactive hydrogen peroxide/oxygen redox couple, there is a growing interest in the use of organometallic polymers in biosensors for electron mediation. These “Type II” sensors were developed (Fig. 1B) by Heller and co-workers,^3^ utilizing a redox polymer as electrochemical mediators rather than oxygen and hydrogen peroxide. Heller was the first to describe an osmium polymer used in a biosensor, which is now considered the most popular strategy for achieving effective electrical wiring of redox enzymes.^3^ Additionally, the high oxidation potential used for the peroxide detection can lead to problems with interferents such as acetaminophen (APAP). APAP can be oxidized near the same potential of the hydrogen peroxide, therefore, if a person had taken acetaminophen and then measured their blood sugar levels, the electrode would not only report on the amount of hydrogen peroxide present (e.g. glucose), but the acetaminophen as well.^4^ The redox active component of these polymers has an E_1/2_ that is lower than the oxidation potential of acetaminophen and therefore would not oxidize acetaminophen at the electrode in the same way as the Type I sensors.^3,5^ Type II sensors are more challenging to develop because synthesizing the organometallic polymer can be time-consuming and difficult, and optimizing the sensor film requires optimization of thickness, loading of redox couple onto a polymeric backbone, and stability lifetime. The stability variables come from the amount of redox polymer, the amount of enzyme, and the amount and type of crosslinker being used.

The redox polymer utilized for the Type II biosensors described in this work^4^ is osmium (II) bis(2,2′-bipyridine) chloride-poly (vinylimidazole-allylamine) (Os(bpy)_2_Cl-PVIAA). This compound is a widely used redox polymer in the field of biosensors.^3–7^ This literature includes the syntheses of the Os(bpy)_2_Cl-PVIAA polymer using an Os(bpy)_2_Cl_2_ precursor to facilitate the loading of the redox couple onto the PVIAA backbone. Most previous literature commonly references the syntheses from Meyer et al. and Buckingham et al., which both use the same protocol.^3,5–9^ These two references detail the use of nitrogen gas purges to provide an oxygen free environment during the intermediate synthesis rather than more rigorous methods, such as a vacuum gas manifold.^10^ Unfortunately, this literature does not include NMR structural analysis or mass spectrometry of the intermediates, but focused on other methods of characterization such as absorbance or E_1/2_ values. More recently, Finklea et al.^11,12^ did include NMR for the Os compounds in their synthetic papers. We note that none of these references observed any oxygen sensitivity.

During our synthesis of this osmium redox polymer for creating new sensors to avoid acetaminophen interference,^4^ we determined that the synthesis of the intermediate Os complex was very sensitive to effects of trace oxygen. The result of trace oxygen exposure to this intermediate resulted in a redox polymer that was no longer useful for the desired type II oxidase biosensor as its redox potential shifted to more positive potentials. Careful consideration using a vacuum gas manifold for the oxygen-free synthesis is important during the Os complex intermediate synthesis, even though the resulting desired Os redox polymer is not particularly oxygen sensitive. Here, we provide detailed protocols for the oxygen-free synthesis of Os(bpy)_2_Cl_2_, along with additional compounds required to create Os(bpy)_2_Cl-PVIAA. Structural, optical, and electrochemical characterizations are given for the desired compounds as well as the oxygen-containing intermediates, which agree with the reported literature values. Based on mass spectrometry, NMR, luminescence, and electrochemical data, we conclude that OsO_2_(bpy)_2_-PVI is the resulting redox polymer from the exposure to trace oxygen during the synthesis.

## Experimental

### Reagents and materials.—

OsCl_3_·3H_2_O (99.95%) was purchased from Strem Chemicals. Dimethylformamide (DMF; 99.9%) and 200- proof ethanol was obtained from Fisher Scientific. ^1^H NMR solvents CDCl_3_, D_2_O and Acetone-d_6_ (99.9%) were obtained from Cambridge Isotope Lab Inc. The following chemicals were obtained from Sigma Aldrich: (NH_4_)_2_OsCl_6_ (99.99%), 2,2’-bipyridine (reagent plus ⩾99%), ethylene glycol (99.9%), anhydrous diethyl ether (99.9%), hydrochloric acid (HCl; 37% w/v), 1—vinylimidazole (⩾99.9%), allylamine (AA, 98%), sodium dithionite (technical grade) and azobisisobutyronitrile (AIBN; 99%).

### Preparation of Os(bpy)_2_Cl_2_.—

Various methods from the literature were used to synthesize the Os(bpy)_2_Cl_2_ precursor; brief descriptions are all found below with the amended first:

From Meyer et al.,^8^ one (1) equivalent of (NH_4_)_2_OsCl_6_ and two (2) equivalents of 2,2’-bipyridine were refluxed in ethylene glycol for 45 min under N_2_. Once cooled, an equi-volume amount of saturated aqueous sodium dithionite solution was added to the reaction mixture to reduce all Os to the 2^+^ oxidation state. The purple-black precipitate was washed with cold water to remove ionic products, and large volumes of ether to remove organic products. As will be shown in results and discussion, oxygen is a more powerful contaminant than previous studies assumed. This synthetic method was performed using a vacuum manifold to minimize the risk of oxygen exposure.

From Finklea et al.,^11,12^ 500 mg of (NH_4_)_2_OsCl_6_ were combined with 350 mg of 2,2’- bipyridine in 10 ml of deoxygenated DMF. The mixture was refluxed under a blanket of N_2_ for an hour. Once the solution was cooled to room temperature, a solution of saturated sodium dithionite reduced any Os(III) to Os(II), and the mixture was cooled further in an ice bath, filtered and dried under vacuum.

From Mamo et al.,^13^ an adaptation of a Ru(bpy)_2_Cl_2_ synthesis from Togano et al.,^14^ 0.5 g of OsCl_3_·3H_2_O was dissolved in 15 ml of 200-proof ethanol and 10 ml DI water and was refluxed with continuous N_2_ bubbling for 4 h. To this solution, 0.7 g of 2,2′-bipyridine dissolved in 10 ml of 200-proof ethanol and 2 ml of conc. HCl was injected and the mixture refluxed for an additional 30 min. The solution was subsequently cooled, the liquid evaporated down, filtered and dried under vacuum.

### Preparation of PVIAA.—

To synthesize poly(vinylimidazole-allylamine), 1.04 ml of 1-vinylimidazole, 0.750 ml allylamine, and 12 ml of 200-proof ethanol were combined in a three neck round bottom flask and purged with N_2_ for 15 min while agitating. During the purging process, a solution of azobisisobutyronitrile (AIBN) was prepared from 65 mg of AIBN and 2 ml of 200-proof ethanol. The AIBN solution was added to the N_2_-purged reaction flask via syringe. The reaction vessel was placed into a preheated 85 °C oil bath. While not typically advised due to the degradation of the AIBN, a slightly better yield was found when the reaction solution was already slightly warm.^15^

### Preparation of Organometallic-PVIAA.—

To synthesize the final polymer, Os(bpy)_2_Cl_2_ and PVIAA were combined in 200-proof ethanol (10–15 ml in a 100 ml round bottom flask) and gently heated using an oil bath to 85 °C, stirring for 48 h with a reflux condenser that was capped to limit ethanol evaporation. During this time the Os(bpy)_2_Cl_2_ was loaded onto the PVIAA backbone under reflux, providing a 1:10 molar ratio Os to imidazole.^3,5^ At the end of 48 h, the reaction mixture was cooled and the ethanol was slowly evaporated using gentle heating and a N_2_-stream until a small volume remained. The remaining solution was diluted with DI water, then centrifuged in order to remove any unreacted organometallic compounds. The water was then divided further among more centrifuge tubes and diluted with more 200-proof ethanol and prepped for lyophilization (see SI (available online at stacks.iop.org/JES/169/016506/mmedia)). The resulting solids could then be dialyzed for a more uniform size, and the same procedure followed of dissolving in water, diluting in ethanol and lyophilizing. This procedure is the same for both organometallic-PVIAA compounds discussed in this paper, and any previous iterations that were used for experimentation.

### Lyophilization procedure.—

Labconco Freezone 4.5 Plus and associated products were used to perform lyophilization on all samples for this project. Products were solvent exchanged or diluted into D.I. water. Solution was then placed inside centrifuge tubes that had holes made in the caps with hypodermic needles. Solution was placed inside of each tube to the corresponding freeze line limit in each. Each tube was then placed inside liquid nitrogen until completely frozen. Once frozen the tubes were placed into the compatible glass jar and rubber cap with glass tube and then placed onto the lyophilizer. Vacuum was then slowly applied. The tubes were periodically observed every 2–8 h depending upon the amount of liquid that was being lyophilized. Once product was finished this process might be repeated if dialyzed.

### Dialysis.—

Spectra/Por Biotech Cellulose Ester MWCO 10 K dialysis membrane was used to perform dialysis. Several inches of membrane were cut from the roll and the end was clasped with a magnetic dialysis clip. The membrane was opened and the fluid poured in and the top was clasped with a non-magnetic dialysis clip to where no air was inside the membrane, only fluid. Foam was rubber banded to the clasps to ensure floatation and the membrane is placed into a 1000–2000 ml container of D.I. water on a stir plate constantly stirring for 18–24 h.

### Instrumentation & Characterization.—

All analyses were performed at an analyte concentration of 1 mg ml^−1^ unless otherwise stated. ^1^H NMR (400 MHz) was performed in CD_3_CN for 2,2′ bipyridine, CDCl_3_ for Os(bpy)_2_Cl_2_, and CO(CD_3_)_2_ for the f-Os precursor. Both osmium organometallic polymers were dissolved in D_2_O for their ^1^H NMR spectra. Mass spectrometry was performed using a Thermo Liquid Chromatography/Mass Spectrometer Orbitrap 2 with electrospray ionization (ESI) in positive mode (Mass Spectrometry Research Center, Vanderbilt University). Fluorescence measurements were performed using a Varian Cary Eclipse fluorescence spectrophotometer with the excitation and emission slit widths set at 5 nm. The scan rate was set to 600 nm per minute with a data interval of 1 nm. Absorption experiments were performed to best determine which excitation wavelengths were suitable. From there, an excitation wavelength was selected to ensure maximum absorption of light, and therefore maximize emission if it was to occur. This is shown and elaborated on in Results and Discussion. Electrochemical measurements were taken using a CH Instruments 620 A Potentiostat and screen-printed electrodes (SPEs) from Pine Research Instrumentation with a 2 mm OD carbon working electrode, a Ag/AgCl reference electrode and carbon counter electrode.

## Results and Discussion

Initial attempts at the synthesis of Os(bpy)_2_Cl_2_ were made following the procedures of Meyer et al., Mamo et al., and Finklea et al., which describe the use of a reflux condenser and N_2_ purging.^8,11–13^ Under these conditions, an unexpected osmium compound was obtained as the major product. The synthesis that provided Os(bpy)_2_Cl_2_ was Meyer et al., but was performed with a vacuum gas manifold to prevent any chance of oxygen contamination during synthesis.

To identify the unexpected Os complex, we employed electrospray ionization mass spectrometry and NMR spectroscopy. The resulting OsO_2_(bpy)_2_-PVIAA was easily identified by its bright luminescence in comparison to Os(bpy)_2_-PVIAA, and a large shift in its oxidation potential seen in the cyclic voltammetry.

### Mass spectrometry.—

**Figure 2.**
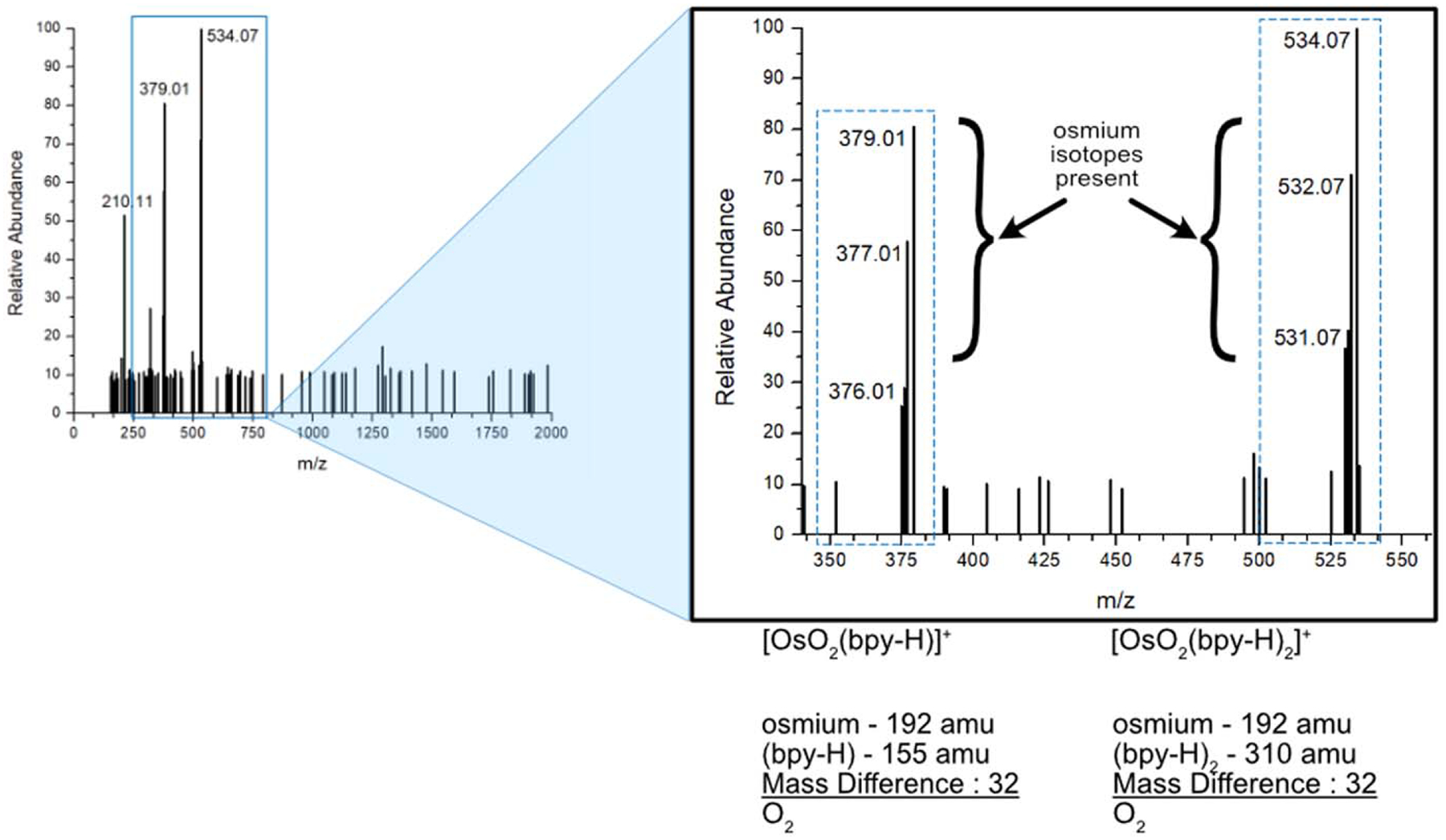
Mass Spectra of f-Os precursor or OsO_2_(bpy)_2_. The full spectrum is shown on the left featuring the main peaks of the molecule, and a zoom in on the right of the parent peak and subpeak at 534.07 and 379.01 m z^−1^ respectively. The descending peaks next to each indicate the osmium isotope being present in each, and the significant weight to these compounds shows that bipyridine needs to be present in each molecule.

The ESI mass spectra of the oxygen-containing Os(bpy)_2_ intermediate complex in shown in Fig. 2. Both the parent peak at 534.07 m z^−1^ and at subpeak 379.01 m z^−1^ have a unique descending isotope spacing in the preceding peaks. This is indicative of osmium’s presence due to its isotopic abundance. When further analyzing the two peaks, we see structural similarities. In the parent peak (534.07 m z^−1^), once we account for the most abundant osmium-192 isotope (~40%) there is considerable mass left to the molecule. First, the base peak is assigned one osmium, and two bipyridines based on isotopic pattern. With that, we are left with a mass difference of 32 amu. In the subpeak (379.01 m z^−1^), following the same process with one osmium-192, and a single bipyridine, we are left with a mass difference of 32 amu. Our results are consistent with a previous mass spectrometric analysis of unsaturated Os complexes with diatomic oxygen by Molina-Svendsen et al.^16^ In our data, the OsO_2_(bpy-H)^+^ was detected at 379 m z^−1^ from the addition of two oxygen atoms and with loss of corresponding hydrogen from the now negatively charged bipyridine from the ESI process. For the base peak at 534.07 m z^−1^, this mass to charge would suggest the OsO_2_(bpy-H)_2_^+^ ion. Thus, we believe the identity of the organometallic that was consistently generated during the oxygen-contaminated synthesis of Os(bpy)_2_Cl_2_, is OsO_2_(bpy)_2_Cl_2_ and [OsO_2_(bpy)]Cl_2_ which would then go on to form OsO_2_(bpy)_2_-PVI and OsO_2_(bpy)-PVI. We will keep using the short hand of f-Os-PVI for OsO_2_(bpy)_2_-PVIAA for the duration of this article, as we will use Os-PVI for Os(bpy)_2_-PVIAA.

### ^1^H NMR.—

**Figure 3.**
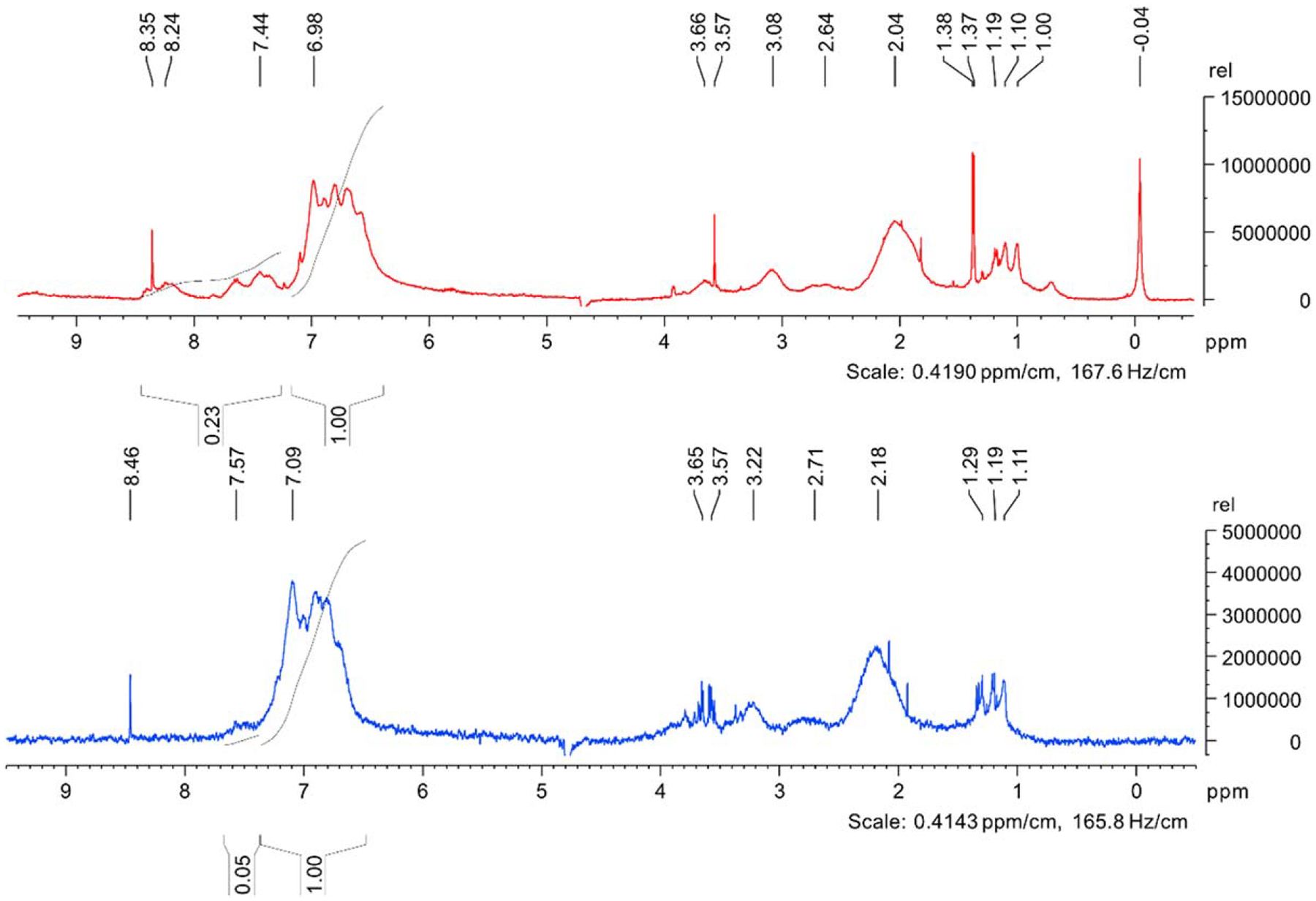
^1^H NMR Spectra Os(bpy)_2_Cl-PVI (Red), and f-Os-PVI (Blue). As shown, the 0–6 ppm shifts the two spectra show the similar characteristics of the PVI backbone. In the ~7 ppm shift range, show the broad peaks of the imidazole side chains. The differences appear from approximately 7.2–8.5 ppm. The Os-PVI shows the downfield shifts of osmium bound imidazole. The f-Os-PVI spectra shows a further shift of the base imidazole peak, due to the oxygen determined to be in the product.

Comparing the proton NMR spectra of the two polymers Os-PVI (red, top) and f-Os-PVI (blue, bottom) allows for direct comparison of their structural similarities and differences. (Fig. 3). Since these compounds are polymers, sharp peaks (e.g. ~8.4 ppm; ones expected in a typical small molecule NMR spectra) are contaminants that were not removed during attempts at purification. Peaks shown in the ~1.1–1.3 ppm range are representative of hydrogens on primary, secondary and tertiary carbons in the backbone of the polymer chain. The broad peak at ~2–2.2 ppm also represents the hydrogens in the backbone of the polymer chain. Since they are almost always in positions that are a carbon removed from aromatic groups they behave as “benzylic,” explaining their ppm shift, higher relative signal, and broadening. The same scenario applying to vinylic hydrogens would explain the small, rounded peak at ~2.6–2.7 ppm. The small number of amino hydrogens on the ends of each polymer strand should account for ~3.5–3.7 ppm. The tallest peak within both spectra is located at ~7 ppm accounts for the aromatic hydrogens on the imidazole groups. Further, the small shifts that have separated from the larger peaks in the Os-PVI spectra following at 7.44 ppm and 8.24 ppm, represent the hydrogens on imidazole groups that have been de-shielded due to the high electron density of osmium coordination at this position.^17^ This indicates Os(bpy)_2_Cl_2_, or rather [Os(bpy)_2_Cl], has bound to the polymer. In the f-Os-PVI spectra, a much smaller peak has follows at 7.57 ppm. The magnitude of this peak suggests that the osmium precursor had not bound as efficiently to the polymer backbone.

Metal loading, the amount of the organometallic precursor bound to the polymer backbone, can be estimated via NMR integrals.^15^ By setting the peak of the imidazole to 1.00, and adjusting the ratios for the osmium shifted peaks accordingly, we can then utilize the following equation to determine the approximate metal loading of the polymer overall.

$$\text{Approximate Metal Loading}=\frac{\text{osmium}-\text{affected proton integrals}}{\text{normalized }\left(\text{to }1\right)\text{imidazole proton integral}+\text{osmium}-\text{affected proton integrals}}\times 100\%$$

For example, during the processing of the ^1^H NMR spectra for Os-PVI, the integrals for the imidazole protons, and the osmium-affected protons were picked. The imidazole peak integral was set to 1.00, and the software automatically adjusted the value of the osmium-affected protons to 0.23. Using these two values, we estimated the metal loading for the batch of Os-PVI utilized in these experiments.

$$\text{Approximate Metal Loading}=\frac{0.23}{1.00+0.23}\times 100\%\approx 20\%$$

To summarize, the Os-PVI has an approximate metal loading of 20% and the f-Os-PVI has an approximate metal loading of 5%. Since the concentrations of the Os-PVI and f-Os-PVI samples were the same we can make direct comparisons between the two. The f-Os-PVI did not load as much osmium onto the polymer backbone as the Os-PVI, according to this NMR, consistent with the extra oxygen coordination around the Os center in f-Os-PVI inhibiting attachment to the PVIAA backbone.

### Fluorescence.—

**Figure 4.**
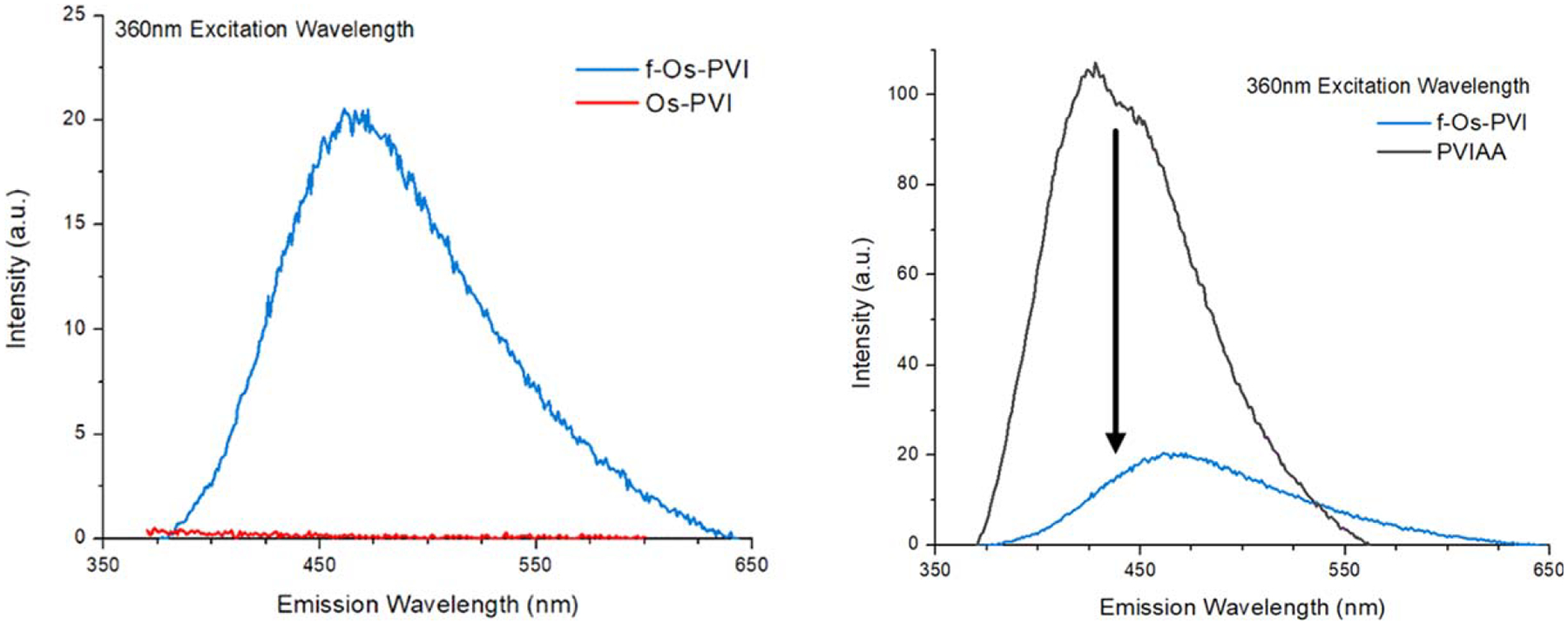
Fluorescence Emission Spectra for PVIAA, Os-PVI, and f-Os-PVI. The Os-PVI (red) does not demonstrate light emission (left). Attaching the f-Os precursor to create f-Os-PVI (blue) quenches the baseline light emission from the PVIAA backbone (black) and shifts the wavelength from approximately 430 nm to 475 nm (right).

Oxygen contamination of the organometallic polymer gave rise to a unique luminescent property not previously seen in Os redox polymers. For the fluorescence of the organometallic polymers when excited in the UV (360 nm) region, the f-Os-PVI was able to emit light in the visible spectrum while the Os-PVI was not (Fig. 4). In comparison to the original PVIAA backbone, the fluorescence has been significantly quenched and slightly red-shifted (by approximately 25 nm). While the quenching data alone supports a lower loading amount of the organometallic onto the polymer backbone, the color shift also confirmed a change in the energetics of the emission.

### Cyclic voltammetry.—

**Figure 5.**
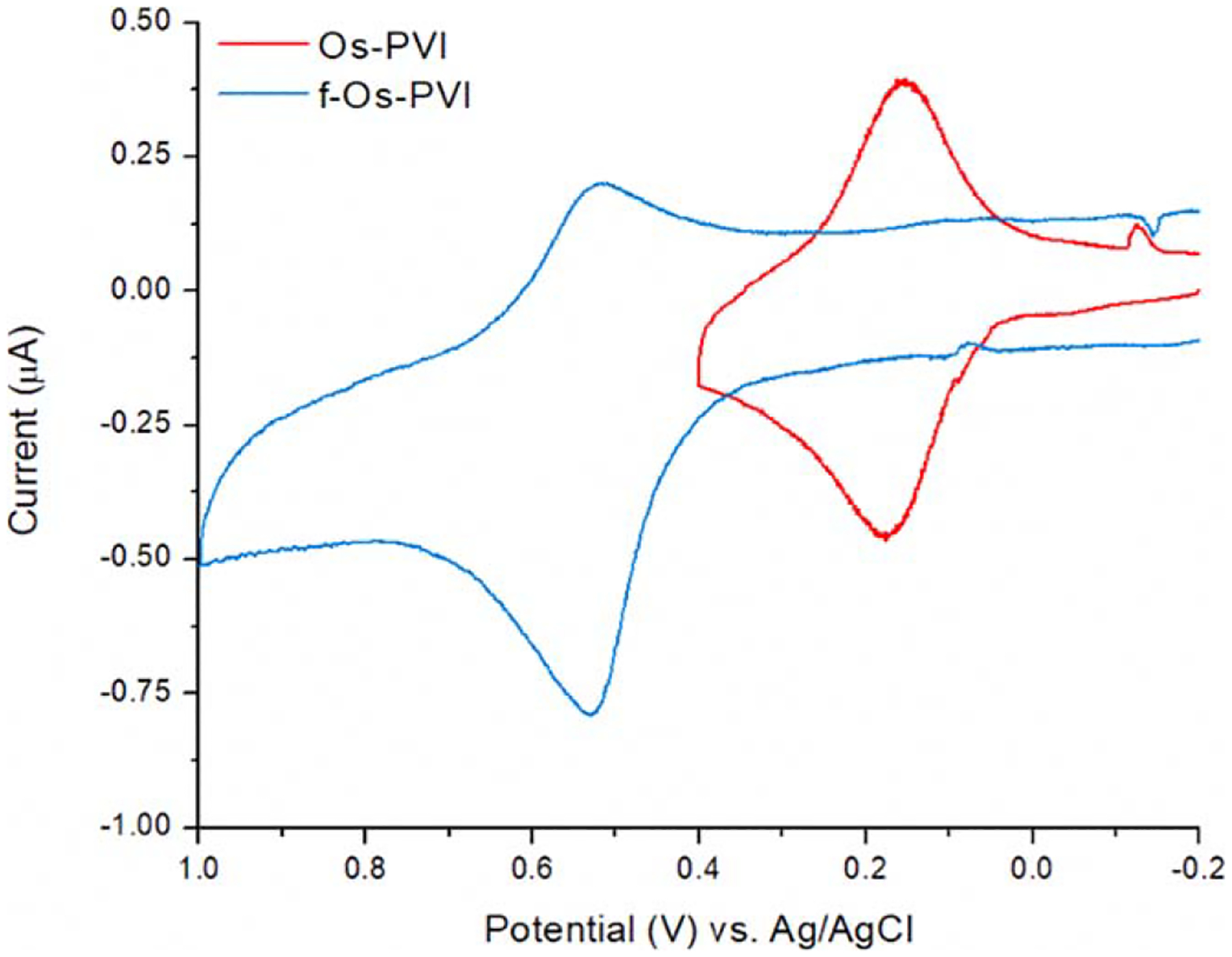
Cyclic Voltammograms of Os-PVI (red) and f-Os-PVI (blue). The cyclic voltammogram characterization for each of the polymers demonstrates their electrochemical reversibility. The Os-PVI i_p_ are approximately equal and the ΔE_p_ suggests electrochemical reversibility. The f-Os-PVI has a distinct difference between the cathodic and anodic current peak heights, providing evidence of ease in electron transfer during oxidation and difficulty during reduction.

The Os-PVI had an E_1/2_ of +0.15 V vs Ag/AgCl, which is expected of this polymer based on literature values and its usefulness in Type II oxidase biosensors.^18^ The NMR and fluorescence data above suggests that the osmium did attach, and this is supported by the CV as the other organic compounds in solution are not redox active. However, attachment of the f-Os precursor to the polymer backbone shifted the E_1/2_ to +0.5 V vs Ag/AgCl, which reflects the oxygen coordination of the Osmium center shifting its oxidation to more positive potentials (Fig. 5). Unfortunately, this large positive shift no longer allows for this redox polymer to be useful for oxidase enzyme-based electrodes, it may be useful for electrochemical mediation for other enzymatic co-factors needing more positive potentials.

Trace oxygen exposure during Os(bpy)_2_Cl_2_ synthesis provides mixed yields of Os(bpy)_2_Cl_2_ along with Os(bpy)_2_O_2_. Once incorporated into the polymer the OsO_2_ polymer has a E_1/2_ that is closer to the hydrogen peroxide E_1/2_ which will reduce the efficiency of the polymer significantly. This makes the OsO_2_ polymer an unsuitable choice for biosensors. Since both compounds will bind to the backbone polymer it is important to minimize oxygen contamination to maximize the end yield of the efficient Osmium polymer. The OsO_2_(bpy)_2_ and f-Os-PVI complexes were stable throughout the course of the characterization and was not subjugated to further stability testing. If a further use of this oxo-containing complex is found, a crystal structure could be pursued to determine its structure.

## Conclusions

We have shown that oxygen contamination for the synthesis of Os(bpy)_2_Cl_2_ will lead to low yields of the desired intermediate, and to a completely different Os complex, OsO_2_bpy_2_Cl_2_. This compound is hard to distinguish from its original counterpart during the synthesis. After mass spectrometry, NMR, luminescence, and electrochemical characterization were distinguished, the role of trace oxygen during the intermediate synthesis was determined. This oxygen-containing complex, and its resulting redox polymers demonstrated reversible electrochemical behavior, but are not able to mediate electron transfer in oxidase enzyme wired electrodes. In the future, this new redox polymer may be useful for electrochemical mediation at its higher potential for other enzyme systems.

## Supplementary Material

supplemental

## Figures and Tables

**Figure 1.**
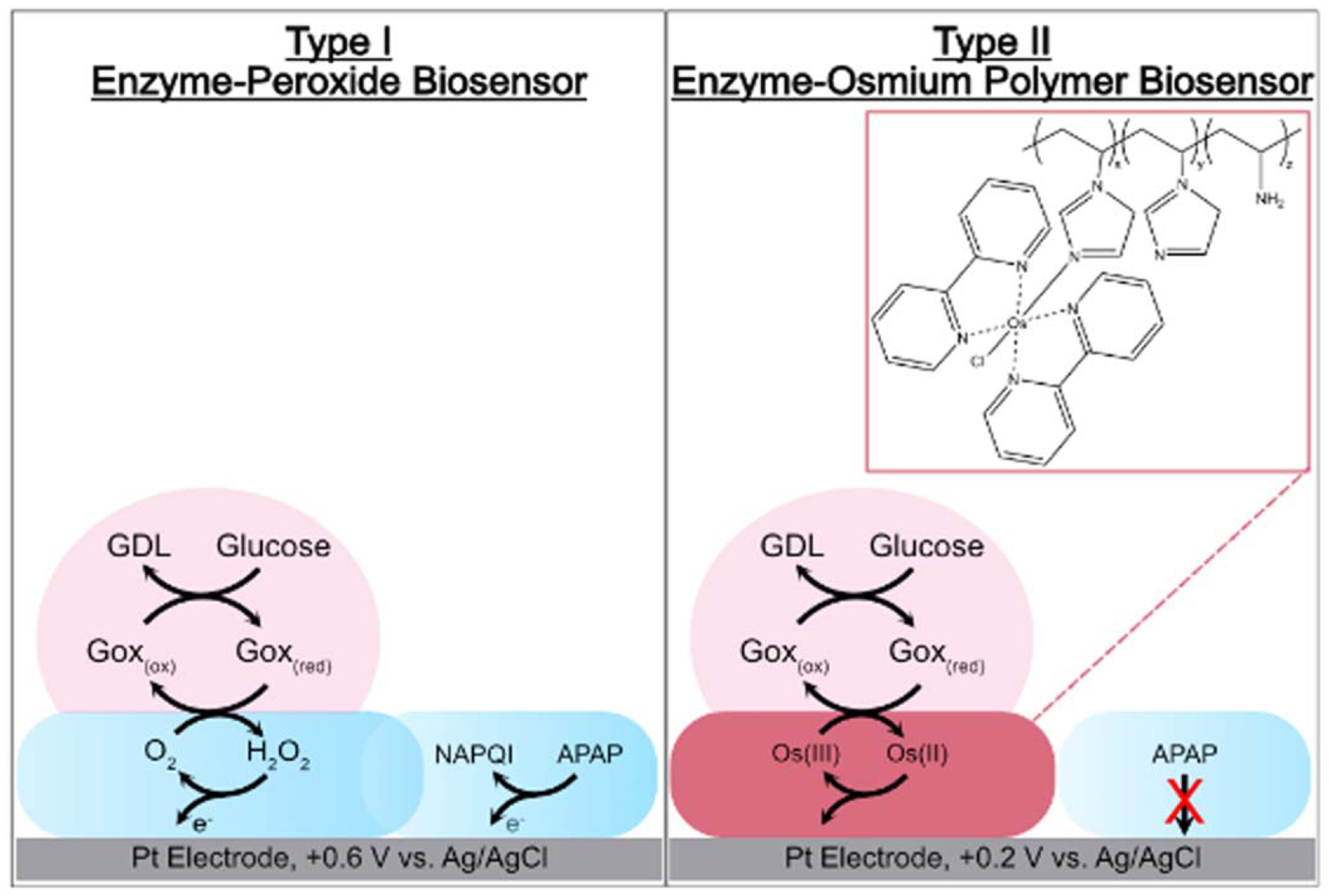
Type I vs Type II Oxidase Enzyme Electrochemical Biosensors: In type I sensors, oxidase enzymes react with dissolved oxygen in solution to produce hydrogen peroxide that is oxidized at the underlying Pt electrode. Type II sensors bypass peroxide by using a redox polymer to accept the electrons from the enzyme.
