# Supplementary material for: Trace Oxygen Affects Osmium Redox Polymer Synthesis for Wired Enzymatic Biosensors
Source: J Electrochem Soc. Author manuscript; Available in PMC 2022 Jun 9. (PMC9183174; doi:10.1149/1945-7111/ac42a0)
Supplement: supplemental [file NIHMS1807715-supplement-supplemental.docx]

Supplemental Information





Supplemental 1. Precursor CVs of Os(bpy)_2_Cl_2_ and the f-Os-Precusor (OsO_2_(bpy)_2_). The unfilled shell around the osmium in the f-Os-Precusor leads to adsorption to the carbon electrode causing high capacitance.


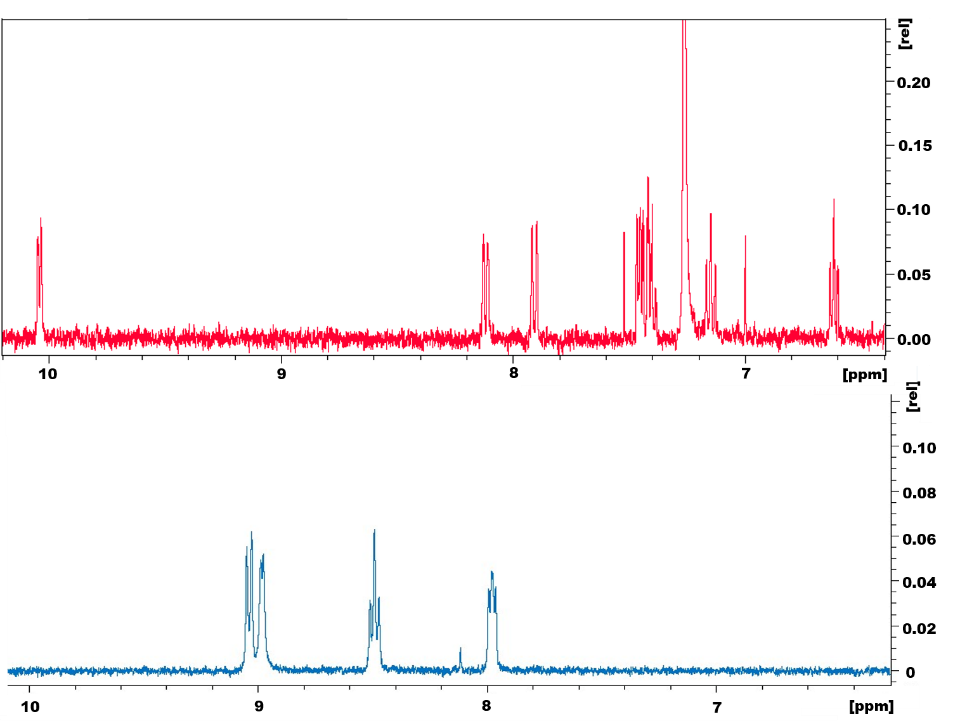


Supplemental 2. Precursor ^1^HNMRs of Os(bpy)_2_Cl_2_ and the f-Os-Precusor (OsO_2_(bpy)_2_). Os(bpy)_2_Cl_2_ demonstrates a large number of peaks expected with the *cis* structure. The f-Os-Precursor shows few peaks suggesting a structure with symmetry.
